# Free from your experiences to grow: belief in free will moderates the relationship between attachment avoidance and personal growth initiative

**DOI:** 10.1186/s40359-023-01289-x

**Published:** 2023-08-24

**Authors:** Fan Yang, Takashi Oka

**Affiliations:** 1 Department of Psychology, College of Humanities and Sciences, Nihon University, 3-chōme-25-40, Sakurajōsui, Setagaya City, Tokyo 156-8550 Japan; 2 Graduate School of Letters, Arts and Sciences, Waseda University, 1-24-1 Toyama, Shinjuku, Tokyo 162-8644 Japan

**Keywords:** Attachment security, Belief in free will, Personal growth initiative, Moderating effects

## Abstract

**Background:**

Attachment theory proposes that attachment security facilitates personal growth. However, attachment security origins in relationship history, and thus, how people treat their experiences may influence the outcomes of attachment security. People differ in the degree in believing that human beings have free will, and belief in free will may influence the relationship between experiences and outcomes. The present cross-sectional study investigated the relationships between attachment security, belief in free will, and personal growth initiative.

**Methods:**

We used the cross-sectional data of 346 Chinese college students for data analysis, including correlational analyses, regression, and moderation analyses. The nine-item Chinese version of the Experiences in Close Relationships-Relationship Structures Scale, the sixteen-item Chinese version of the Personal Growth Initiative Scale-II, and the seven-item Free Will subscale of the Chinese version of the Free Will and Determinism Plus Scale were utilized.

**Results:**

Results showed attachment avoidance and belief in free will, not attachment anxiety, was associated with personal growth initiative. Belief in free will moderated the association between attachment avoidance and personal growth initiative. When the centered score of belief in free will was higher than 0.64, attachment avoidance was no longer associated with personal growth initiative. 85.84% of our data were below this Johnson-Neyman significance region, and 14.16% were above. In other words, only those who scored higher than 0.64 on free will beliefs were able to pursue personal growth despite their high attachment avoidance.

**Conclusions:**

These findings suggest that when believing in free will, avoidantly attached people may believe in their ability to pursue personal growth and think their future has more possibilities, not influenced by other factors like social support, which they think they lack.

## Background

Attachment theory was initially developed to describe and explain the affectional bond formed between an infant and caregivers during the early years of life, and this affectional bond is stored via internal working models, which contain memories regarding the interactions with caregivers [1]. Ainsworth and Bell [2] developed a strange situation paradigm to classify different attachment styles of infants. Meanwhile, they noticed that the attachment figure not only serves as a safe haven that can support infants when they are in need but also as a secure base from whom infants start to explore the environment with confidence. Hazan and Shaver [3] applied attachment theory to adulthood and claimed that the three categories, that is, secure, avoidant, and anxious/ambivalent, are roughly the same as in infancy. In contrast, attachment styles are related to the models of self and social relationships. Bartholomew and Horowitz [4] proposed a four-category model of adult attachment based on the dimension of self-model and other-model, and Griffin and Bartholomew [5] further confirmed the construct validity of self-and-other-model dimensions underlying the four-category model of adult attachment. Brennan et al. [6] categorized adult attachment strategies into two dimensions, namely, attachment anxiety and attachment avoidance. Those who score highly in attachment anxiety tend to adopt hyperactivating strategy when threatened, while those who score highly in avoidance tend to adopt deactivating strategy when threatened [7]. A lack of both attachment anxiety and avoidance represents a high attachment security [8, 9]. Research from social psychology tends to use attachment anxiety and avoidance as two indicators of attachment security, and low levels of both attachment anxiety and avoidance represent attachment security. The former four categories could be regarded as combinations of different levels of attachment anxiety and avoidance [8]. Evidence supports that two dimensions describe attachment-related individual differences better than four categories [6, 8, 10, 11]. According to the adult attachment theory [8], attachment security not only functions as a safe base that provides individuals with support and comfort, but more importantly, it also plays the role of security base, which could facilitate personal growth initiative. Since attachment security is malleable [12–15], exploring the relationship between attachment security and personal growth initiative may provide insights for future interventions. Moreover, the current study also examined the condition under which attachment security matters. Specifically, belief in free will was supposed to moderate the association between attachment security and personal growth initiative.

### The relationship between attachment security and personal growth initiative

Pursuing personal growth and its motivation has long been regarded as an important topic in psychological science [16, 17]. However, little is known regarding the relationship between attachment security and personal growth. According to the broaden-and-build cycle of attachment security [18–21], because of their successful experiences of coping with distress and the consequent positive emotions, securely attached individuals (low scores in both attachment anxiety and attachment avoidance) are prone to be more resilient [22–25] and to explore the world initiatively. Attachment security could motivate individuals to seek growth, and the attachment behavior system could activate the exploring behavior system. Personal growth initiative (PGI) is conceptualized as an active, intentional engagement in the process of personal growth, consisting of the cognitive and behavioral components [26]. Robitschek [27] proposed a four-dimension model of the personal growth initiative, including readiness, planfulness, using of resources, and intentional action. Readiness for change refers to the preparedness for making specific changes for themselves. Planfulness refers to the ability to make effective personal growth-related plans. Using resources refers to taking advantage of available resources to facilitate personal growth. Intentional behavior refers to pursuing personal growth consciously.

Some studies focus on factors facilitating personal growth initiative, including the A-B type personality [28] and family functioning [29]. Previous studies do not find an association between age and personal growth initiative [26, 30]. However, there is currently no study investigating the relationship between attachment security and personal growth initiative. We predicted that a higher level of attachment avoidance, not anxiety, could be associated with lower personal growth initiative. Individuals with a high level of attachment avoidance tend to avoid asking others for help and neglect their feelings. Moreover, pursuing personal growth could sometimes be challenging, and avoidant people may keep their distance from potential threats to maintain independence. Therefore, those with high attachment avoidance may have lower personal growth initiative. In contrast, individuals with a high level of attachment anxiety show ambivalent attitudes and behaviors. The effects of attachment anxiety on psychological outcomes tend to be counterbalanced by these ambivalent attitudes and behaviors [31], leading to a weak or insignificant relationship between attachment anxiety and personal growth initiative. Previous research found that although anxiously attached people may be curious, they fear their curiosity may harm their interpersonal relationships [32]. Moreover, attachment avoidance, but not anxiety, could negatively predict support seeking [33], which is important to personal growth initiative. Therefore, we hypothesized that attachment avoidance could negatively affect personal growth initiative. Still, attachment anxiety may have a small or insignificant correlation with personal growth initiative, consistent with previous research [31]. Though there might be an association between attachment avoidance and personal growth initiative, belief in free will may moderate this association.

### The moderating role of belief in free will

The relationship between attachment avoidant and personal growth initiative may be moderated by belief in free will. Attachment is one of the human behavior systems, and non-attachment variables could impact the outcomes of attachment. Attachment mainly origins in relationship history, and thus how one treats their experiences may impact the influence of attachment. One important factor is whether people believe they are self-determined or determined by other factors, that is, belief in free will. Whether people believe they have free will may play a role in the relationship between attachment and its outcomes. Belief in free will means believing that in a given situation, one could choose to behave differently regardless of experiences instead of behaving in a specific way [34]. Belief in free will could predict future behaviors, such as focusing more on outcomes than actions, cheating behaviors, and helping behaviors (for a review, see Ewusi-Boisvert & Racine, [35]). A recent review found that decreasing belief in free will affects implicit and explicit feelings of self-control and perceived self-control and decreases learning from emotional experiences [35]. Previous research pointed out that people’s concept of free will consists of three components: a) the alternative possibility, b) the ability to make one’s own decisions, and c) acting without internal or external constraints [36]. Those who believe in free will may thus take different actions from the past, re-thinking the influence given by experiences and exploring more possibilities. When believing in free will, avoidantly attached people may strongly believe in their ability to pursue personal growth on their own and think their future has more possibilities, not influenced by other factors like social support, which they think they lack. Thus, attachment avoidant may have less impact on personal growth initiative when believing in free will. Moreover, according to the self-determination theory (SDT; Ryan & Deci. [37]), when behaviors are perceived to be self-determined, instead of being determined by other factors, individuals will act more autonomously, leading to a higher level of personal growth initiative. This suggests that belief in free will may positively correlate with personal growth initiative. We hereby hypothesize that belief in free will could moderate the association between attachment avoidance and personal growth initiative. More specifically, for those who score highly in belief in free will, the association between attachment avoidant would be stronger than others.

Based on the above theoretical understandings and hypotheses, we decided to conduct a preliminary study to explore the relationship between attachment avoidance and personal growth initiative and the moderating role of belief in free will in this relationship. The current study is the first to explore the relationship between attachment security and personal growth initiative, and the moderating role of belief in free will in this relationship. Through this exploration, we hope to provide preliminary evidence for the secure base function of attachment security. In other words, a sense of attachment security facilitates people to focus on personal growth, whereas attachment anxiety and avoidance hamper personal growth. Personal growth initiative is associated with many beneficial outcomes, such as lower distress and higher wellness [38]. Since attachment security (indicated by attachment anxiety and avoidance) could be primed [39] and can be changed [12–14], it thus provides a possibility that facilitating individuals’ personal growth initiatives via reducing their attachment avoidance. Moreover, the current study also contributes to adult attachment theory by attempting to explore the condition of the impact of attachment security by examining the moderating effect of belief in free will in the association between attachment avoidance and personal growth initiative. The purposes of the present study were to examine the three hypotheses a) belief in free will positively correlates with personal growth initiative; b) attachment avoidance significantly negatively correlates with personal growth initiative; c) belief in free will moderates the relationship between attachment avoidance and personal growth initiative.

## Method

The present study utilized a cross-sectional design, and data in the present study are available from the authors on reasonable request.

### Participants

We conducted an online survey and delivered our measures through Chinese social networks. Before participation, the respondents familiarized themselves with the privacy policies of the study. They were informed that they would be considered willing to participate if they chose to complete and submit the survey. Three hundred and eighty-seven participants completed the measures mentioned below. However, only 346 participants (45.4% males, 54.0% females, 0.6% others; *M*_age_ = 21.710 years, *SD* = 2.198 years) correctly answered attention check tests, being regarded as valid data. We conducted the interaction power analysis using the InteractionPoweR Shiny App (https://david-baranger.shinyapps.io/InteractionPoweR_analytic/). Our results suggest that our interaction power was above 80% with a significance level of α = .05. This implies that if the interaction effect we are predicting exists, there is more than an 80% probability that we would detect it in our data.

### Measures

#### Demographic variables

Gender (male, female, and others) and age were collected as the demographic variables. Demographic variables were controlled when conducting regression analysis.

#### Attachment security

The nine-item Chinese version of the Experiences in Close Relationships-Relationship Structures Scale (ECR-RS) developed by Peng et al. [15] was adopted to measure adult attachment in the present study. Participants were required to answer the items on a 7-point scale (1 = not at all, 7 = completely true). The ECR-RS contains two subscales: attachment anxiety (e.g., “I worry that my dating partner won't care about me as much as I care about him or her”; α = .87) and avoidance (e.g., “I prefer not to show my dating partner how I feel deep down”; α = .79). Mean scores were calculated for attachment anxiety and avoidance. A higher mean score represents a higher level of attachment anxiety or avoidance, respectively. A lower score on both attachment anxiety and avoidance represents a higher level of attachment security.

#### Personal growth initiative

The sixteen-item Chinese version of the Personal Growth Initiative Scale-II (CPGIS-II) developed by Yang et al. [40] was adopted to measure personal growth initiative. CPGIS-II (α = .94) includes four subscales, namely, readiness (e.g., “I can tell when I am ready to make specific changes in myself.”; α = .83), planfulness (e.g., “I set realistic goals for what I want to change about myself.”; α = .86), using of resources (e.g., “I ask for help when I try to change myself.”; α = .74), and intentional action (e.g., “I take every opportunity to grow as it comes up.”; α = .84). Participants were required to answer the items on a 7-point scale (1 = not at all, 7 = completely agree). The mean score was calculated for the personal growth initiative. A mean higher score means a higher level of personal growth initiative, readiness, planfulness, using of resources, or intentional action, respectively.

#### Belief in free will

The seven-item Free Will subscale of the Chinese version of the Free Will and Determinism Plus Scale (FAD+) developed by Liu et al. [41] was adopted to measure belief in free will. This subscale measures the degree to which participants strongly believe in free will (e.g., “People have complete control over the decisions they make.”; α = .75). The mean score was calculated for belief in free will, with a higher mean score suggesting a stronger belief in free will.

#### Attention check test

Considering that the attitudes and attention of participants could be unstable during the period of filling the measurement [42], two attention check tests were included in order to make sure the validation of data and to recognize the random answers. The attention check tests were respectively ‘I answered some questions in the present study without reading them’ and ‘Please choose 3’. A recent study [43] supports the idea that it is reasonable to believe that these attention check tests will not cause serious problems on scale validity.

#### Data analysis

Data analysis was conducted using IBM SPSS Statistics Version 21, and Model 1 of the PROCESS Macro for SPSS Version 3.5 [36] was used to examine the moderating effect. We made reference to previous research [30, 44] and included age and gender as covariables. Considering the current study is a preliminary study, we also conducted correlational analyses to examine the associations between attachment anxiety, attachment avoidance, belief in free will, and personal growth initiative before conducting the moderation analysis. Moreover, though we did not make specific predictions regarding the subscales of personal growth initiative, we also included them in correlational analyses as an exploring analysis, as it may be helpful for future studies.

## Results

### Descriptive statistics

Table 1 reports the means and standard deviations. Table 2 reports the correlations among the main variables in the present study. Attachment avoidance significantly negatively correlated with belief in free will, personal growth initiative, and all subscales of personal growth initiative. Attachment anxiety did not significantly correlate with personal growth initiative, belief in free will, and any subscale of the personal growth initiative.

**Table 1:** Descriptive statistics for variables used in the present study (*N* = 346)

Variables	*M*	*SD*
1. Attachment Anxiety	3.99	1.62
2. Attachment Avoidance	2.77	1.14
3. Belief in Free Will	3.70	0.66
4. Personal Growh Initiative	4.39	0.74
5. Readiness	4.23	0.91
6. Planfulness	4.30	0.84
7. Using of Resources	4.53	0.81
8. Intentional Action	4.50	0.79

**Table 2:** Correlations among the main variables in the present study (*N* = 346)

	1	2	3	4	5	6	7
1. Attachment Anxiety	-						
2. Attachment Avoidance	.00	-					
3. Belief in Free Will	.07	-.14**	-				
4. Personal Growth Initiative	.08	-.34***	.44***	-			
5. Readiness	.06	-.25***	.36***	.89***	-		
6. Planfulness	.08	-.27***	.42***	.94***	.85***	-	
7. Using of Resources	.02	-.39***	.35***	.78***	.53***	.62***	-
8. Intentional Action	.08	-.33***	.42***	.92***	.72***	.81***	.72***

^**^p < .01

^***^p < .001 (2-tailed)

### Conditional process model

**Fig. 1:**
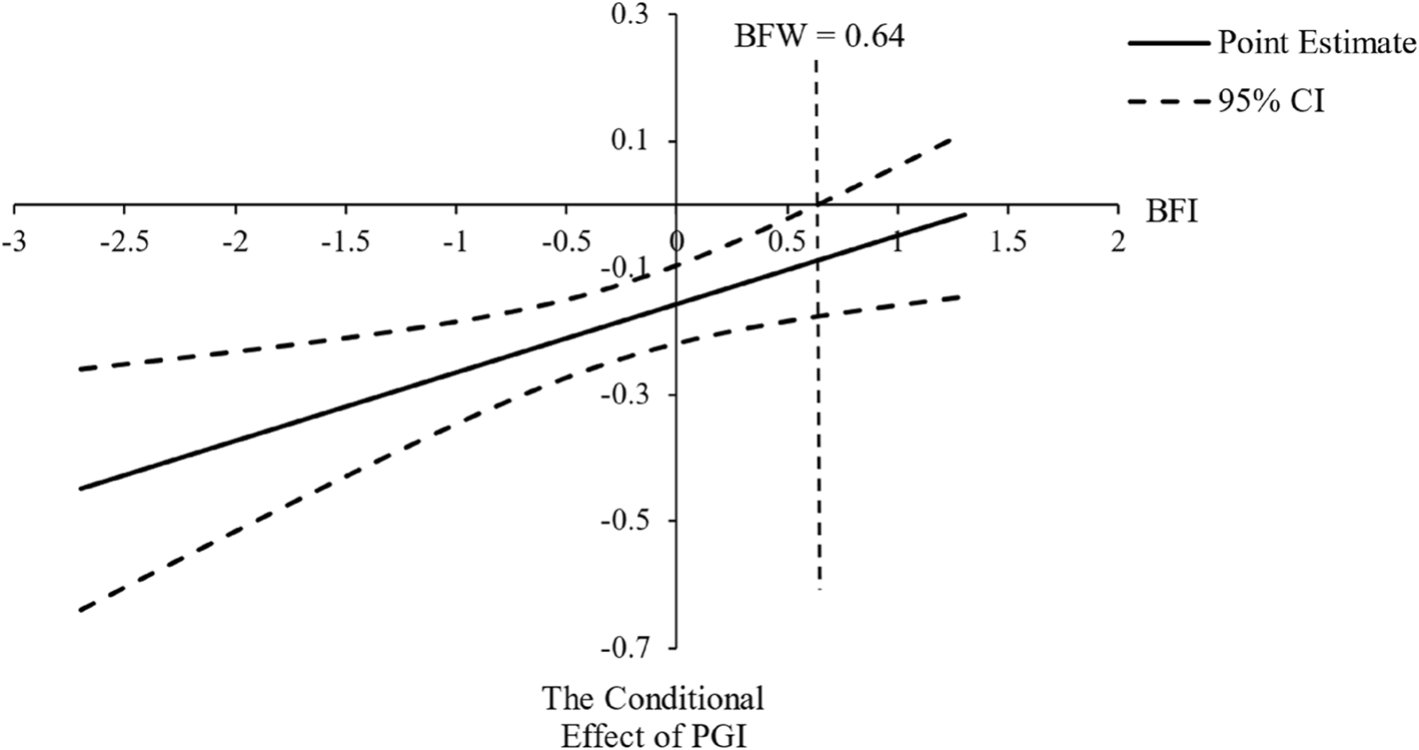
The moderating effect of belief in free will on the relationship between attachment avoidance and personal growth initiative (*N* = 346). PGI = Personal growth initiative; BFW = Belief in free will

We conducted the regression model with attachment avoidance and belief in free will as independent variables, the interaction of attachment avoidance and belief in free will as a moderator, personal growth initiative as the dependent variable, and gender and age as control variables (see Table 3) to test the conditional process model [45]. Firstly, after controlling age and gender, attachment avoidance predicted personal growth initiative. Belief in free will significantly predicted personal growth initiative. After controlling age and gender, attachment avoidance, belief in free will, and the interaction of attachment avoidance and belief in free will accounted for 31% of the total variance of personal growth initiative. The interaction of attachment avoidance and belief in free will was also significantly associated with personal growth initiative, demonstrating the moderation effect of belief in free will between attachment avoidance and personal growth initiative (see Fig. 1). We applied the Johnson-Neyman technique for testing the moderation effect [46, 47], which enabled us to identify regions in the range of the moderator variable where the effect of the independent variable on the outcome was significant and not significant. The independent variable was attachment avoidance, the moderator was belief in free will, and the dependent variable was personal growth initiative. This analysis yielded a statistically significant moderation effect (see Table 3). When a centered score is higher than 0.64, a value defined by the Johnson-Neyman significance region [47], the conditional effect of attachment avoidance on personal growth initiative transitions to non-significance (see Fig. 1). That is, the relationship between attachment avoidance and personal growth initiative was only significant for those who scored lower than 0.64 on belief in free will. 85.84% of our data was below this Johnson-Neyman significance region, and 14.16% was above.

**Table 3:** The regression models of variables in present study (*N* = 346)

Dependent Variable	Independent Variables	*R*^2^	*F*	*B*	*SE*	*t*	LLCI	ULCI
Personal Growth Initiative	Age	.31	30.40	-.02	.02	-1.42	-.05	.01
	Gender			-.20	.07	-2.95**	-.33	-.07
	Attachment Avoidance			-.16	.03	-5.05***	-.22	-.10
	Belief in Free Will			.43	.05	8.17***	.33	.54
	Interaction			.11	.04	2.88**	.03	.18

Interaction = Attachment Avoidance * Belief in Free Will

*LLCI* Lower Limit of Confidence Interval, *ULCI* Upper Limit of Confidence Interval

^**^p<.01

^***^p<.001 (2-tailed)

## Discussion

Personal growth initiative is a valuable positive psychological disposition and is related to a range of positive outcomes, such as self-esteem, self-efficacy, self-compassion (for a review, see de Freitas et al. [48]), mental health (for a review, see Weigold et al. [38, 49]). We tested the relationships among attachment anxiety, attachment avoidance, belief in free will, and personal growth initiative to explore the determinants of personal growth initiative and the way of facilitating personal growth initiative. Results showed that attachment anxiety did not significantly correlate with any other variables in the present study, including belief in free will, personal growth initiative, and all subscales of the personal growth initiative. Attachment avoidance and belief in free will were independently associated with personal growth initiative. This relationship was moderated by belief in free will.

Attachment anxiety did not correlate with attachment avoidance and belief in free will. Indeed, attachment anxiety and avoidance were supposed to be orthogonal [6, 8], and therefore their correlation may be small or insignificant. With regard to the belief in free will, it may be a belief that is not directly linked to the attachment system because it is not specified in interpersonal relationships or threatening contexts, which is important for the attachment system. Though there was a significant correlation between attachment avoidance and belief in free will, it could be regarded as weak in research on individual differences [50], and thus we tended to interpret it cautiously, which requires further studies.

These results were consistent with prior research that attachment anxiety did not significantly correlate with personal growth and attachment avoidance negatively correlated with personal growth [51]. When pursuing personal growth, it is important to seek help from friends and take advantage of attainable resources. However, according to Simpson et al. [31], behaviors of highly anxious people may reflect behavioral ambivalence in which tendencies to both approach and withdraw from the partner effectively counterbalance one another. Moreover, theoretically, attachment anxiety may have two-sided relationships with personal growth initiative. This aligns with a previous study suggesting that, on the one hand, ambivalent persons desired to explore the world, described themselves as curious, and engaged in information search. On the other hand, they believed that they should not be as curious as they were and that curiosity could jeopardize relationships [32]. These could lead to an insignificant correlation between attachment anxiety and personal growth initiative (as well as its subscales). Avoidantly attached individuals hold negative views of others and do not believe in the kindness of others. Therefore, avoidantly attached individuals tend to solve problems independently and even refuse the help offered by friends or partners, which would hamper their personal growth initiative. Indeed, previous research found attachment avoidance, but not anxiety, made a significant unique contribution to predicting support seeking [33].

We tested whether belief in free will was associated with personal growth initiative and the moderation effect of belief in free will on the relationship between attachment avoidance and personal growth initiative. Results demonstrate that belief in free will was positively associated with personal growth initiative. This finding supports the perspective of self-determination theory (SDT; Ryan & Deci. [37]) that the sense of being able to make choices and being autonomous caused by belief in free will is related to positive psychological outcomes, leading to growing initiatively. We also found that when highly believing that free will exists, attachment avoidance was no longer associated with personal growth initiative. This finding is congruent with the idea that how adult individuals interpret their experiences is important in the relationship between attachment and psychological outcomes [52]. Furthermore, when adding the interaction of attachment avoidance and belief in free will, the association between personal growth initiative and belief in free will was no longer statistically significant. This may imply that the effect of belief in free will was mainly through the interaction with attachment avoidance. Belief in free will means people tend to believe there are alternative possibilities, they have the ability to make their own decisions, and their behaviors may not be determined by internal or external factors [36]. Avoidantly attached people deal with stress by keeping their distance, while pursuing personal growth could be challenging. Though they may realize that social support is necessary for achieving personal goals, they do not think that others will help them get through when faced with challenges and frustration. However, when they strongly believe in free will, they may be open to new possibilities and think they can achieve goals without other factors, such as social support.

There are several limitations existing in the present study. Firstly, we only used a cross-sectional design, so causal relationships could not be inferred. Future studies need to manipulate the level of belief in free will and test the relationship between attachment, belief in free will, personal growth initiative, and the moderation effect with experimental methods. Secondly, we only used the Free Will subscale of the Chinese Version of Free Will and Determinism Plus Scale (FAD+). However, this prevents us from drawing a whole picture of the moderating effect of free will-related beliefs toward the relationship between attachment avoidance and personal growth initiative. Thus, future studies need to test the effects of other free will-related and determinism-related beliefs on attachment and personal growth initiative. Thirdly, though the previous study showed that in Western countries, there were surprising patterns in people’s intuitions about free will and moral responsibility [53], the understanding of free will may vary in Eastern countries, and researchers also cast doubts on the universality claim of attachment theory [54], and therefore the effects of belief in free will on attachment and personal growth initiative need to be tested with samples of different cultures. Moreover, alternative explanations, such as the implicit theory [55, 56], should be examined in future studies as well. There are differences between belief in free will and implicit theory. Individuals with a low belief in free will may think personal growth is determined by experiences, family, or random factors, but they could still think their personal growth is still malleable and changeable. However, theoretically, both the belief in free will and the implicit theory relate to the way people think of their own power in personal growth. Thus, it would be interesting for future research to compare their effects on personal growth.

Despite these limitations, the current study provides the first preliminary evidence regarding the relationship between attachment avoidance and personal growth initiative and the moderating effect of belief in free will. This is consistent with the secure base hypothesis that a lack of attachment security would hamper personal growth [8], emphasizing that a history of secure relationship experience may contribute to not only interpersonal outcomes but also the positive development of oneself. Considering that attachment avoidance could be reduced by priming or other interventions [12–14, 57], it may be viable to facilitate personal growth initiatives by reducing attachment avoidance. The moderating effect of belief in free will suggests that though past experience matters, how individuals treat their experience may alter the impact of the past. Therefore, the current study provides preliminary evidence for the marginal condition of the impact of attachment security. This marginal condition of the impact of attachment security is of theoretical significance. Researchers have pointed out that efforts should be paid to clarify not only what attachment is and its influences but also what it should not and should not directly influence [58]. Clearer demarcation of the boundary conditions of attachment constructs and processes will help researchers more effectively build and interpret the next generation of attachment findings, including those that are ambiguous or inconsistent in relation to the theory [58]. Moreover, practically, since belief in free will can be manipulated [35, 59], it may be helpful for facilitating personal growth initiative via cultivating a belief in free will, especially for those with high attachment avoidance.

## Conclusion

The present study indicated that belief in free will was positively associated with personal growth initiative, attachment avoidance was negatively associated with personal growth initiative, and belief in free will moderated the association between attachment avoidance and personal growth initiative. Specifically, for those who scored highly in belief in free will, attachment avoidance could not significantly predict their personal growth initiative.

## Data Availability

The datasets used and/or analyzed during the current study are available from the corresponding author upon reasonable request.

## Abbreviations

ECR-RS: The nine-item Chinese version of the Experiences in Close Relationships-Relationship Structures Scale
CPGIS-II: The sixteen-item Chinese version of the Personal Growth Initiative Scale-II
FAD+: The seven-item Free Will subscale of the Chinese version of the Free Will and Determinism Plus Scale
BFW: Belief in Free Will
PGI: Personal Growth Initiative
