# Correction to: Free from your experiences to grow: belief in free will moderates the relationship between attachment avoidance and personal growth initiative

**DOI:** 10.1186/s40359-023-01304-1

**Published:** 2023-09-05

**Authors:** Fan Yang, Takashi Oka

**Affiliations:** 1 Department of Psychology, College of Humanities and Sciences, Nihon University, 3-chōme-25-40, Sakurajōsui, Setagaya City, Tokyo 156-8550 Japan; 2 Graduate School of Letters, Arts and Sciences, Waseda University, 1-24-1 Toyama, Shinjuku, Tokyo 162- 8644 Japan

**BMC Psychology (2023) 11:243**

10.1186/s40359-023-01289-x

Following publication of the original article [1], the authors flagged that the association between attachment avoidance and personal growth initiative had been misrepresented in the Conclusion: where it says “attachment avoidance was negatively associated with personal growth initiative”, it previously (erroneously) said “attachment avoidance was positively associated with personal growth initiative”. The original article has since been updated to correct this error, and the authors thank you for reading this erratum and apologize for any inconvenience caused. 

